# Revision of Palearctic species of the genus *Dimorphaphorura* (Collembola: Onychiuridae: Onychiurinae: Oligaphorurini) with description of new species

**DOI:** 10.1093/jis/14.1.74

**Published:** 2014-01-01

**Authors:** Wanda M. Weiner, Igor J. Kaprus’

**Affiliations:** 1 Institute of Systematics and Evolution of Animals, Polish Academy of Sciences, Sławkowska 17, Pl-31016 Kraków, Poland; 2 State Museum of Natural History, Ukrainian National Academy of Sciences, Teatral’na St. 18, UA-79008 L’viv, Ukraine

**Keywords:** chaetotaxy, geographical distribution, identification key, Palearctic region, taxonomy

## Abstract

In this paper, the Palearctic genus *Dimorphaphorura*Bagnall, 1949 (Collembola: Onychiuridae), is revised. The diagnosis of the genus is defined within the tribe Oligaphorurini based on the development of the furcal area, shape of furcal rudiment, and furcal chaetotaxy. Six new species are described: *D. olenae***sp. n.** from Ukraine, *D. inya***sp. n** . *, D. pseudoinya***sp. n.***, D. sibirica***sp. n.***, D. caucasica***sp. n.** , and *D. sophyae***sp. n.** from Russia. The type species of the genus, *D. differens*Bagnall, 1949 is redescribed, and the lectotype and paralectotypes are designated. All previously known species are redescribed or with additional characters complemented: *D. alnus* ( Fjellberg, 1987 ) **comb. n.** , *D. chatyrdagi* ( Kaprus’, Weiner & Pomorski, 2002 ) **comb. n.** , *D. daii* ( Pomorski, Skarżyński & Kaprus’, 1998 ) **comb. n.** , *Dimorphaphorura eremia* ( Kaprus’,Weiner & Pomorski, 2002 ) **comb. n.** , *D. hackeri* ( Christian, 1986 ) **comb. n.** , *D. irinae* ( Thibaud & Taraschuk, 1997 ) **comb. n.** , *D. melittae* ( Christian, 1993 ) **comb. n.** , *D. pseudoraxensis* ( Nosek & Christian, 1983 ) **comb. n.** , *D. raxensis* ( Gisin, 1961 ) **comb. n.** , *D. steposa* ( Kaprus’, Weiner & Pomorski, 2002 ). An identification key to all *Dimorphaphorura* species is provided.

## Introduction

Bagnall (1949) established the genus *Dimorphaphorura* with the type species *D. differens* for the Tirolian specimens determined and briefly described by Stach (1947) as *Onychiurus quadrituberculatus* (Bőrner, 1901). The scant diagnoses presented by Bagnall (1949) do not allow for the clear differentiation of *Dimorphaphorura* from the genera *Oligaphorura* and *Micraphorura* created by him in the same paper. For this reason, the three genera have been considered junior synonyms of *Onychiurus* Gervais, 1841. Only in 1996 did Pomorski re-established *Oligaphorura* and *Micraphorura* to the generic level, and Weiner (1996) provided new diagnoses for these two genera and *Dimorphaphorura* based on new characters, but she wrongly interpreted the chaetotaxy of the manubrial rows, as she joined chaetae on rows mm and ma (1+1), which she considered as dental chaetae.

Following the authors’ genus level investigations of Onychiuridae, a corrected diagnosis of *Dimorphaphorura* is presented based on type material of described species. The generic status of *Dimorphaphorura* is discussed in light of the consideration made by Shvejonkova and Potapov (2011) . In addition, a list of species recognized as belonging to the genus is given, and the lectotype and paralectotypes for the species studied are designated. Eleven species belonging to the genus *Dimorphaphorura* are currently known from the Palearctic region, distributed across mountainous regions (Tirol, Crimea Mts.), caves (Lower Austria, Crimea), and steppe and forest-steppe (Ukraine). These species were originally described in the genera *Onychiurus* , *Onychiurus* ( *Oligaphorura* ), or *Micraphorura* by Christian (1986 , 1993), Fjellberg (1987) , Gisin (1961) , Kaprus’ et al. (2002) , Nosek & Christian (1983) , Pomorski et al. (1998) and Thibaud & Taraschuk (1997) . Three further species were recently described ( Shvejonkova & Potapov 2011 ; Sun & Wu 2012a , b).

The known species of *Dimorphaphorura* studied based on type material are (repository of type material in parentheses):

*Dimorphaphorura alnus* ( Fjellberg, 1987 ) **comb. n.** : 7 paratypes (TUM)

*Dimorphaphorura chatyrdagi* ( Kaprus’, Weiner & Pomorski, 2002 ) **comb. n.** : holotype and paratype (SNHM)

*Dimorphaphorura daii* ( Pomorski, Skarżyński & Kaprus’, 1998 ) **comb. n.** : 5 paratypes (ZIWU)

*Dimorphaphorura differens* Bagnall, 1949 : lectotype and 3 paralectotype (ISEA)

*Dimorphaphorura eremia* ( Kaprus’, Weiner & Pomorski, 2002 ) **comb. n.** : holotype and 2 paratypes (ZIWU)

*Dimorphaphorura hackeri* ( Christian, 1986 ) **comb. n.** : 2 paratypes (NHMW)

*Dimorphaphorura irinae* ( Thibaud & Taraschuk, 1997 ) **comb. n.** : holotype and 2 paratypes (MNHN)

*Dimorphaphorura melittae* ( Christian, 1993 ) **comb. n.** : 6 paratypes (E. Christian’s collection) *Dimorphaphorura pseudoraxensis* ( Nosek & Christian, 1983 ) **comb. n.** : holotype (MNH)

*Dimorphaphorura raxensis* ( Gisin, 1961 ) **comb. n.** : 2 paratypes (MNH)

*Dimorphaphorura steposa* ( Kaprus’, Weiner & Pomorski , 2002 ) **comb. n.** : holotype and paratype (SNHM)

The recently described species are: *Dimorphaphorura stojkoae* ( Shvejonkova & Potapov, 2011 ) **comb. n.**

*Dimorphaphorura sanjiangensis* Sun & Wu, 2012

*Dimorphaphorura jingyueensis* Sun & Wu, 2012

The new described species are:

*Dimorphaphorura caucasica* **sp. n.**

*Dimorphaphorura inyae* **sp. n.**

*Dimorphaphorura olenae* **sp. n.**

*Dimorphaphorura pseudoinyae* **sp. n.**

*Dimorphaphorura sibirica* **sp. n.**

*Dimorphaphorura sophyae* **sp. n.**

The material studied is deposited in the following institutions:

ISEA – Institute of Systematics and Evolution of Animals, Polish Academy of Sciences, Cracow, Poland;

MNH – Museum of Natural History, Geneva, Swiss;

NHMW – Naturhistirisches Museum, Wien, Austria;

MSPU – Moscow State Pedagogical University, Russia;

SIEE – Severtsov Institute of Ecology and Evolution Russian Academy of Sciences, Moscow, Russia;

SNHM – State Natural History Museum of Ukrainian National Academy of Sciences, L’viv, Ukraine;

TUM – Tromsø University Museum, Department of Natural Science, Norway

ZIWU – Department of Biodiversity and Evolutionary Taxonomy, Zoological Institute, Wrocław University, Poland.

## Materials and Methods

The specimens of the species hereby described were extracted from soil and litter samples using Berlese funnels and stored in 90% ethanol. They were cleared in Amann’s lactophenol and mounted on slide in MarcAndré or Faure’s solution. The type material from the collections were studied and redescribed.

The morphological characters used follow Fjellberg (1999) , Pomorski (1996 , 1998), and Weiner (1996) .

### Nomenclature

This publication and the nomenclature it contains have been registered in ZooBank. The LSID number is:

http://urn:lsid:zoobank.org:pub:AD9A913A-78E5-49A4-A5DC-F1CDD5538706

It can be found online by inserting the LSID number after ( www.zoobank.com/

## Results

### *Dimorphaphorura* Bagnall, 1949 : 510

Type species by original designation (see Weiner 1996 , p. 174): *Dimorphaphorura differens*Bagnall, 1949

*Diagnosis*

Postantennal organ with 1 vesicle divided into 3–4 lobes, in elongated depression, its length about 1.0–1.5 times the diameter of the nearest pseudocellus. Sensory clubs of antenna III sense organ granulated, ribbed or smooth, external one bigger than internal one. Head dorsally with chaeta d0 absent. Labral formula: 4/3, 4, 2. Labial type ABC, AC or A (types after Fjellberg 1999 ). Posterior part of head usually with 2+2 pseudocelli, thoracic tergum I with 0-1+0-1, abdominal terga IV–V with 3-5+3-5 and 3-4+3-4 pseudocelli respectively. Thoracic tergum I with 10 to 15 chaetae. Furcal rudiment (on abdominal sternum IV) with fine granulated area (dental area) and three rows of manubrial chaetae behind its posterior edge (on manubrial area): row ma with 4 (rarely 2) chaetae (ma) (dental chaetae absent), row mm with only 2 external chaetae and row mp with 4–5 chaetae (the external ones = macrochaetae) ( Figure 3 ). Chaetae s on body distinct or only slightly differentiated. Tibiotarsi with 5 to 11 acuminated chaetae in distal whorl. Abdominal tergum VI with chaeta a0 present or absent and p0 present. Spines or spiniform chaetae present or absent.

*Remarks*

**Figure 1-3.**
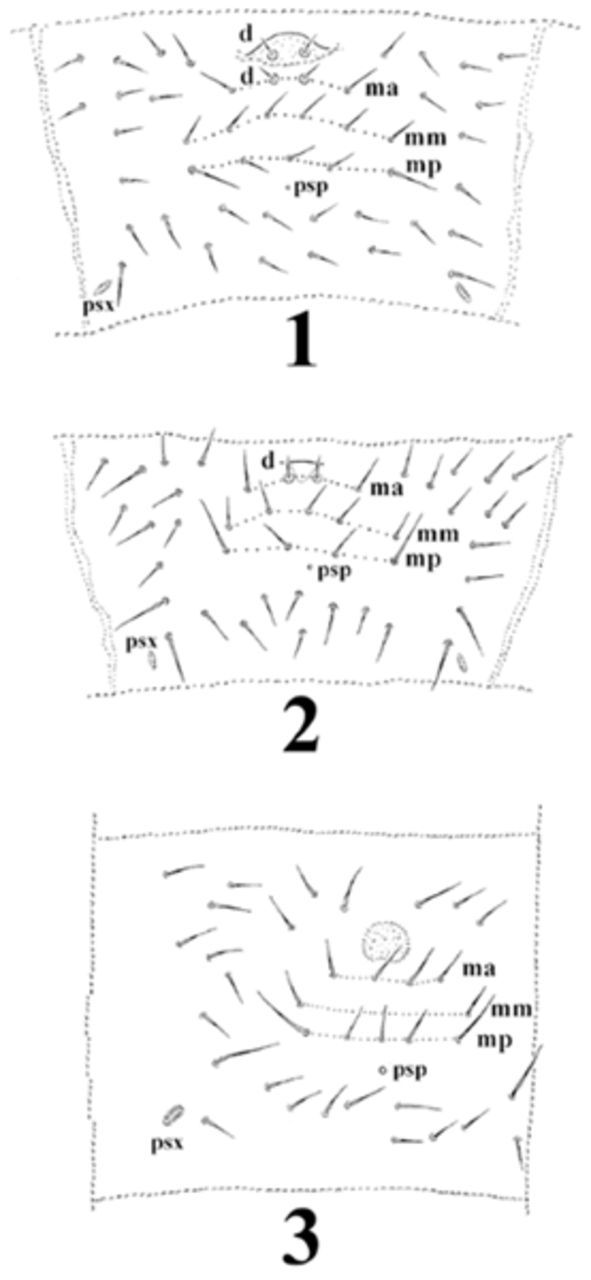
Сentral part of abdominal sternite IV: **1** , *Oligaphorura tuvinica* Potapov & Stebaeva, 1997; **2** , *Micraphorura absoloni* (Börner, 1901); **3** , *Dimorphaphorura differens*Bagnall, 1949 ; d – dental chaetae, ma – anterior row of manubrial chaetae, mm – medial row of manubrial chaetae, mp – posterior row of manubrial chaetae, psp – pseudoporus, psx –parapseudocellus. High quality figures are available online.

Genus *Dimorphaphorura* is most similar to genera *Micraphorura* and *Oligaphorura* . All mentioned genera differ clearly by the organisation of the furcal area, as is presented in Figures 1–3 . *Micraphorura* and *Oligaphorura* possess 1+1 and 2+2 (in two rows) dental chaetae (= setulae according Pomorski 1996 , 1998), respectively, showing special basis ( Figure 1 and 2 ). These chaetae could be accompanied by 1+1 manubrial chaetae (ma). *Dimorphaphorura* is devoid of dental chaetae. Further differation concerns the chaetotaxy of the manubrial area. In *Micraphorura* and *Oligaphorura* , chaetae of manubrial row ma migrated anteriorly to the level of dental chaetae. In addition to chaetae ma, species of these two genera always carry 2 or more medial chaetae in rows mm and mp. In *Dimorphaphorura* , the chaetotaxy of the manubrial area consists of three rows: ma, with 4 (rarely 2) chaetae placed below dental area; row mm, which preserves only 1+1 external chaetae; and row mp, with 4–6 chaetae (external ones as macrochaetae). The dental area is developed differently in all three genera. In *Oligaphorura* , the dental area is developed as a cuticular fold (like in *Protaphorura* ), in *Micraphorura* as a cuticular furrow or rather triangular pocket, and in *Dimorphaphorura* as a fine granulated area. Also, all 11 tibiotarsal chaetae are present in the distal whorl of *Micraphorura* and *Oligaphorura* species, whereas in *Dimorphaphorura* their number could be reduced (11 to 5 chaetae) ( Table 1 ).

**Table 1.**
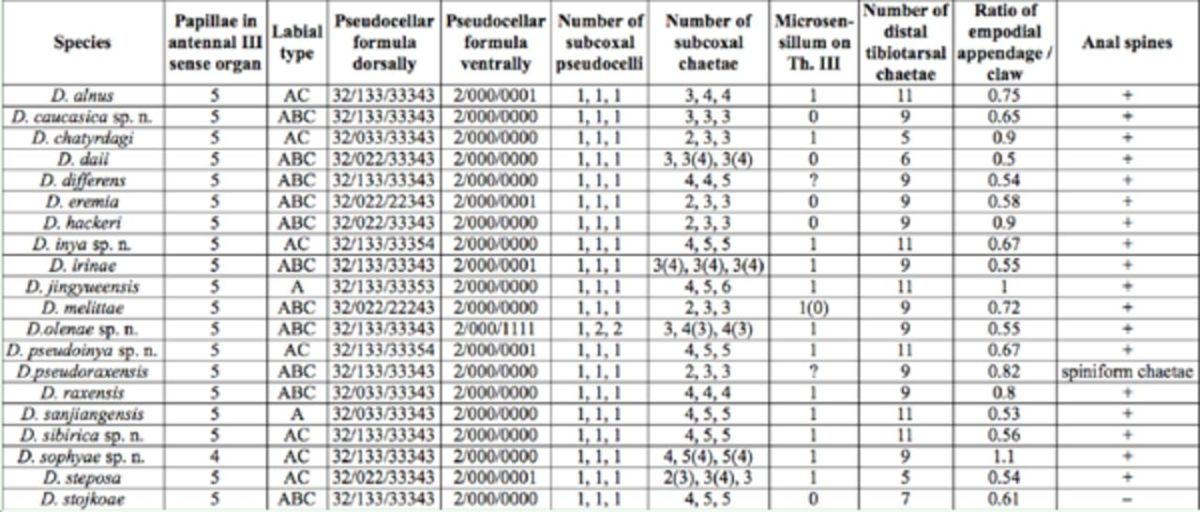
Main diagnostic characters of the known species of *Dimorphaphorura.*

### Description and redescription of species

*Dimorphaphorura differens* **Bagnall, 1949**

( Figures 4–10 )

*Dimorphaphorura differens* Bagnall, 1949 : 511 (= *Onychiurus quadrituberculatus*Stach, 1947 , nec Börner 1901)

*Type material*

Lectotype ♂ on slide, Austria: Tirol, Katterriegel, Haller Mauern, + 1900 m alt., 17.X.1940 leg. H. Franz. Paralectotypes, 3 specimens (sex undetermined) on three slides: Admont X 26/I. det. J. Stach as *Onychiurus quadrituberculatus* . Type repository: ISEA.

*Redescription*

**Figure 4-10.**
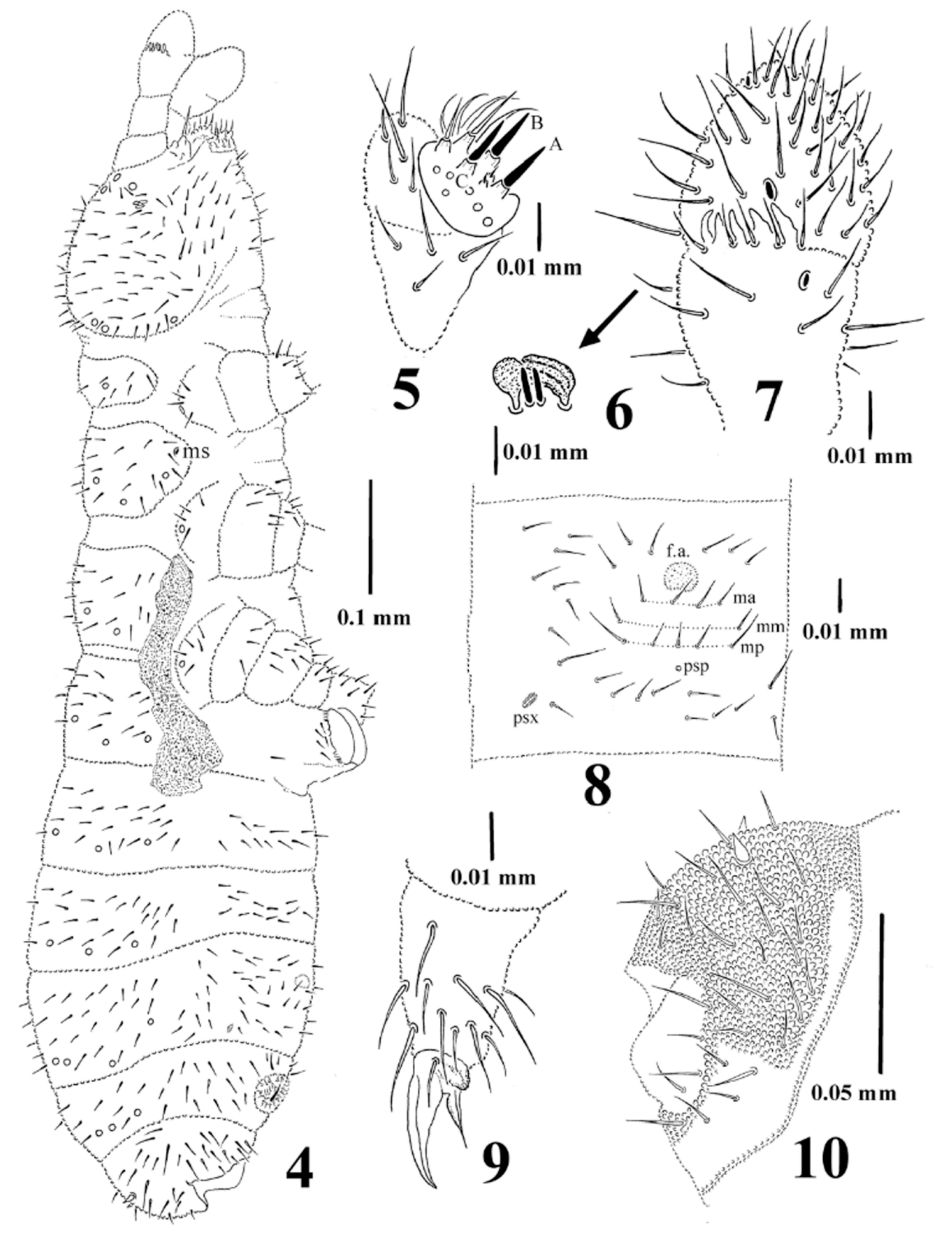
*Dimorphaphorura differens* Bagnall, 1949 : **4** , body chaetotaxy; **5** , labial palp; **6** , sensory clubs and sensory rods; **7** , antennal segments III and IV with antennal III sense organ; **8** , central part of abdominal sternite IV, ms – microsensillum, f.a. – furcal area; **9** , tibiotarsal chaetotaxy and claw of leg III; **10** , abdominal segment VI. High quality figures are available online.

Lectotype length 0.92 mm, length of paralectotypes: 0.83–0.98 mm. Shape of body cylindrical ( Figure 4 ). Colour in alcohol white. Granulation homogenous, with coarse granules on abdominal tergum VI ( Figure 10 ) and head.

Antennal base well marked by finer and regular granulation. Antennae nearly as long as head. Sensory organ of antennal segment III consisting of 5 chaetae, 5 papillae, 2 smooth sensory rods, 2 weakly granulated sense clubs (internal clubs straight and globular, external clubs bigger, weakly ribbed and bent), ventrolateral sensillum present ( Figure 7 ). Antennal segment IV with subapical organite and microsensillum ( Figure 7 ).

Postantennal organ vesicle with three lobes, located in small cuticular depression, as long as 1.5 nearest pseudocellus. Labral chaetotaxy not seen. Maxillary outer lobe with simple palp, sublobal hairs not seen. Labial type ABC ( Figure 5 ).

Pseudocellar formula dorsally: 32/133/33343 ( Figure 4 ), ventrally: 2/000/0000. Parapseudocelli on abdominal sterna I–III not seen. Abdominal sternum IV with 1+1 parapseudocellus ( Figures 4 , 8 ). All subcoxa 1 with 1 pseudocellus; parapseudocelli not seen. Dorsal chaetotaxy symmetrical, as in Figure 4 . Chaetae relatively short, poorly differentiated into macrochaetae and microchaetae. Sensory chaetae s on body distinct, distributed on half tergum as: 2/011/22211. Thoracic tergum I with 7+7 chaetae. Thoracic tergum II with lateral microsensilla. Microsensilla on thoracic tergum III not seen. Abdominal tergum IV with medial chaeta m0. Subcoxa 1 of I–III legs with 4, 4, and 5 chaetae respectively. Chaetotaxy of abdominal sternum IV as in Figures 4 , 8 . Thoracic sterna I–III with 0+0, 1+1, 1+1 chaetae respectively. Ventral tube with 5+5 chaetae, and 1+2 chaetae at base.

Furcal rudiment: fine granulated area and three rows of chaetae behind its posterior edge. Row ma with 4 chaetae, mm with 2 external chaetae and mp with 5 chaetae ( Figure 8 ).

Tibiotarsi I, II, III with 18, 18, 17 chaetae respectively. Distal tibiotarsal whorl with 9 chaetae. Claw without denticles. Empodial appendage shorter than claw (0.54 of inner edge of claw), with distinct basal lamella ( Figure 9 ).

Anal spines 0.48 of length of inner edge of claw and 2.5 times as long as their basal diameter.

### Remarks

*Dimorphaphorura differens* is very similar to *D. caucasica* sp. n., the other mountain species from North Caucasus ( Table 1 ). Both species have the same pseudocellar formula, the same number of chaetae in the distal tibiotarsal whorl, and an antennal sensory organ with 5 papillae. They differ in the number of chaetae on subcoxae 1 (4, 4, and 5 in *D. differens* and 3, 3, and 3 in *D. caucasisa* ), the granulation of the abdominal tergum VI (coarse, in the shape of a band in *D. differens* and homogenous in *D. caucasica* ) and in the size of anal spines (smaller in *D. differens* ).

*Distribution*

Austria: Tirol and Admont.

*Dimorphaphorura alnus* **( Fjellberg, 1987 )** **comb. n.**

*Onychiurus alnus* Fjellberg, 1987 : 282.

*Type material*

Seven paratypes on slide, Russia: Magadan Region, Aborigen, 27.VII.1979, deep, moist *Pinus pumila* litter, leg. A. Fjellberg. Type repository: TUM.

*Other material*

Russia: Chukotka, Chaun Bay, Loc. S12, Sept. 1975, 3 ♀♀ on slides, leg. MacLean, (TUM: TSZX 21174); Wrangel Island (SW), Mamontovaya river Valley, southern slope, herbaceous tundra, 23–24.VII.1994, 3 ♀♀ on slides, leg. A. Babenko; North-Eastern Yakutia, delta of the Kolyma River, tussock tundra, 1994, ♀ on slide, leg. A. Babenko.

*Redescription*

Body length 0.8–0.9 mm. Shape of body cylindrical. Colour white. Granulation homogenous, with coarse granules around all dorsal pseudocelli and on abdominal tergum VI.

Antennae approximately as long as head. Antennal segment I with 8 chaetae, antennal segment II with 12 chaetae. Sensory organ of antennal segment III with 5 chaetae, 5 papillae, 2 smooth sensory rods, and 2 granulated sense clubs (internal clubs straight and globular, external ones bigger, weakly ribbed and bent – not seen well on paratypes), ventrolateral sensillum present. Antennal segment IV with subapical organite and microsensillum.

Postantennal organ vesicle with 3–4 lobes, located in a small cuticular depression, 1.5 as long as nearest pseudocellus. Labral formula of chaetae: 4/3, 4, 2. Maxillary outer lobe with simple palp and 2 sublobal hairs. Labial type AC.

Pseudocellar formula dorsally: 32/133/33343, ventrally: 2/000/0001. All subcoxa 1 with 1 pseudocellus. Parapseudocelli not seen.

Dorsal chaetotaxy symmetrical, chaetae relatively short, poorly differentiated into macrochaetae and microchaetae. Sensory chaetae s on body distinct, distributed on half tergum as 2/011/222211. Thoracic tergum I with 5-6+5-6 chaetae. Thoracic terga II–III with lateral microsensilla. Abdominal tergum IV with or without medial chaeta m0. Abdominal tergum VI with medial chaeta a0. Subcoxa 1 of legs I–III with 3, 4, 4 chaetae respectively. Thoracic sterna I–III with 0+0, 1+1, 1+1(2) chaetae respectively. Ventral tube with 5-6+5-6 chaetae, and 1-2+1-2 chaetae at base.

Furcal rudiment: fine granulated area and three rows of chaetae behind its posterior edge. Row ma with 4 chaetae, row mm with only 2 external chaetae, and row mp with 4 chaetae.

Tibiotarsi I–III with 20, 20, 19 chaetae respectively. Distal tibiotarsal whorl with 11 chaetae. Claw without denticle. Empodial appendage shorter than claw (0.75 of inner edge of claw), with clear basal lamella.

Anal spines 0.74–0.85 of length of claw inner edge and 2.7 times as long as their basal diameter.

*Distribution*

Russia: North-Eastern Siberia.

*Dimorphaphorura caucasica* **Weiner & Kaprus’, sp. n.** ( Figures 11–18 )

*Type material*

Holotype ♀ on slide, Russia: North Caucasus, Karachaevo – Cherkesia, nearby Teberda, Malaya Hatipara Mt., 2750 m alt., alpine meadow, 2.VI.1981, leg. T. Dobrolubova. Paratypes: 4 ♀♀ on slides, the same data as holotype. Type repository: SNHM – holotype and paratypes: 3 ♀♀, ISEA – paratype: 1 ♀.

*Description*

**Figure 11-18.**
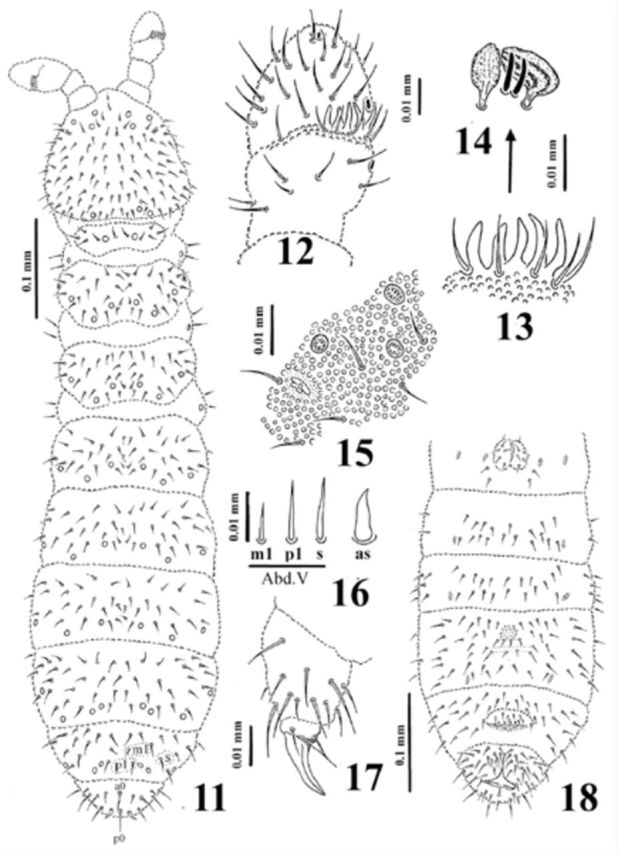
*Dimorphaphorura caucasica* **sp. n.** : **11** , dorsal body chaetotaxy; **12** , antennal segments III and IV with antennal III sense organ; **13** , papillae and guard chaetae of antennal III sense organ; **14** , sensory clubs and sensory rods; **15** , postantennal organ and pseudocelli at base of antenna; **16** , ordinary chaetae (m1, p1) and chaeta s on abdominal tergum V, and anal spine (as); **17** , tibiotarsal chaetotaxy and claw of leg III; **18** , chaetotaxy and localization of parapseudocelli on abdominal sterna I-VI. High quality figures are available online.

Holotype length 0.69 mm, length of paratypes: 0.70–0.74 mm. Shape of body cylindrical ( Figure 11 ). Colour in alcohol white. Granulation homogenous, with coarse granules around all dorsal pseudocelli. Usually 11 grains around each pseudocellus.

Antennae almost as long as head. Antennal segment I with 8 chaetae, antennal segment II with 13 chaetae. Sensory organ of antennal segment III consisting of 5 guard chaetae, 5 papillae, 2 smooth sensory rods, 2 granulated sense clubs (internal clubs straight and globular, external ones bigger, ribbed and bent) ( Figures 12–14 ), ventrolateral microsensillum present. Antennal segment IV with subapical organite and microsensillum ( Figure 12 ).

Postantennal organ vesicle with three lobes, located in small cuticular depression and 1.2 as long as nearest pseudocellus ( Figure 15 ). Labral formula of chaetae: 4/3, 4, 2. Maxillary palp simple with 2 sublobal hairs. Labial type ABC.

Pseudocellar formula dorsally: 32/133/33343, ventrally: 2/000/0000 ( Figures 11 , 18 ). Parapseudocellar (psx) formula ventrally: 1/000/211101 (on anal valves psx unpaired) ( Figure 18 ). All subcoxae 1 with 1 pseudocellus and 1 parapseudocellus.

Dorsal chaetotaxy symmetrical, as in Figure 11 . Chaetae relatively short, weakly differentiated into macro-and microchaetae. Sensory chaetae s present, their formula per half tergum: 2/011/11111. Thoracic tergum I with 5-6+5-6 chaetae. Thoracic tergum II with lateral microsensilla, thoracic tergum III without microsensilla. Abdominal tergum IV without medial chaeta m0. Abdominal tergum VI with medial chaeta a0 and p0. Shape and length of some ordinary chaetae, sensory chaeta s on (abdominal tergum V) and of anal spines in Figure 16 . Subcoxae 1 of legs I–III with 3 chaetae each. Thoracic sterna I–III with 0+0, 1+1, 1+1 chaetae respectively. Ventral tube with 6+6 chaetae, and 2+2 chaetae at base. Chaetotaxy of abdominal sternum IV as in Figure 18 .

Furcal rudiment: fine granulated area and three rows of chaetae behind its posterior edge. Row ma with 4 chaetae, row mm with only 2 external chaetae, and row mp with 4 chaetae ( Figure 18 ).

Tibiotarsi I–III with 18, 18, 17 chaetae respectively. Distal tibiotarsal whorl with 9 chaetae. Claw without denticle. Empodial appendage with small basal lamella and 0.65 of inner edge of claw ( Figure 17 ).

Anal spines 0.69 times of inner claw edge and 2.4 times as long as their basal diameter.

*Remarks*

See remarks in *D. differens* and Table 1

*. Etymology*

The name of the new species refers to the type locality that belongs to the Caucasus Mts.

*Distribution*

Russia: North Caucasus.

*Dimorphaphorura chatyrdagi* **( Kaprus’, Weiner & Pomorski, 2002 ) comb. n.**

*Micraphorura chatyrdagi* Kaprus’, Weiner & Pomorski, 2002 : 359.

*Type material*

Holotype ♀ on slide: Ukraine, Crimean Mts, Chatyr-Dag Mt., Bezdonka Cave, on piece of wood, 140 m from entrance, 6.VI.1993 leg. R. Vargovitsh. Paratype ♀ on slide: the same data as holotype. Type repository: SNHM.

*Other material*

Ukraine, Crimean Mts, Chatyr-Dag Mt., Gigerdzhii cave, on the surface of water, 10.VII.1997, 3 ♀♀, leg. R. Vargovitsh.

*Additions to the original description*

Labial type AC. Maxillary outer lobe with simple palp and with 2 sublobal hairs. Sensory chaetae s on body slightly differentiated. Subcoxa 1 of legs I–III with 2, 3, 3 chaetae respectively. Furcal rudiment consisting of a fine granulated area and three rows of chaetae behind its posterior edge. Row ma with 2–3 chaetae arranged asymmetrically, row mm with 2 external chaetae and row mp with 4 chaetae. Empodial appendage with large basal lamella and 0.9 of claw inner edge.

*Distribution*

Ukraine: Crimean Mts.

*Dimorphaphorura daii* **( Pomorski, Skarżyńki & Kaprus’, 1998 ) comb. n.** *Micraphorura daii* Pomorski, Skarżyńki & Kaprus’, 1998 : 253.

*Type material*

Paratypes: 4 ♀♀and 1 ♂, Ukraine: Crimea Mts., neighbourhood of Jalta, Nikitskij Pereval, ca. 1450 m a.s.l., litter and grasses on mountain meadow 12.IX.1997, leg. R.J. Pomorski, D. Skarżyński & I.J. Kaprus’). Type repository: ZIWU.

*Additions to the original description*

Postantennal organ 2.0 times as long as nearest pseudocellus. Labral formula: 4/3, 4, 2. Maxillary outer lobe with simple palp and 2 sublobal hairs. Labial type ABC. Thoracic tergum I with 5-6+5-6 chaetae. Subcoxa 1 of legs I–III with 3, 3(4), 3(4) chaetae respectively. Furcal rudiment includes a fine granulated area and three rows of chaetae behind its posterior edge. Row ma with 2 chaetae, row mm with 2 external chaetae, and row mp with 4 seate. Tibiotarsi I–III with 14, 14, 13 chaetae respectively. Distal tibiotarsal whorl with 6 chaetae. Anal spines 0.54 times inner claw edge and 2.5 times as long as their basal diameter.

*Distribution*

Ukraine: Crimea Mts.

*Dimorphaphorura eremia* **(Kaprus’, Weiner & Pomorski, 2002) comb. n.**

*Micraphorura eremia* Kaprus’, Weiner & Pomorski, 2002: 354.

*Type material*

Holotype ♂ and 2 paratypes: 1 ♂ and juvenile on slides, Ukraine: Podolia, near Stara Ushycia, National Park of Podolian Tovtry, moss and wet soil near stream, decidous forest on calcareous rocks, Bakota, 9.XI.2001, leg. R.J. Pomorski. Type repository: ZIWU.

*Additions to the original description*

Sense clubs in sensory organ of antennal segment III slightly granulated (internal clubs straight and globular, external ones bigger, ribbed, and bent). Labral formula of chaetae: 4/3, 4, 2. Maxillary palp simple with 2 sublobal hairs. Labial type ABC. Pseudocellar formula dorsally: 32/022/22343 (in original description 32/022/22333). Thoracic tergum I with 4-5+4-5 chaetae. Subcoxa 1 of legs I–III with 2, 3, 3 chaetae respectively. Furcal rudiment comprises a fine granulated area and three rows of chaetae behind its posterior edge. Row ma with 4 chaetae, row mm with 2 external chaetae, and row mp with 4 chaetae. Empodial appendage with small basal lamella.

*Distribution*

Ukraine: Podillya Region.

*Dimorphaphorura hackeri* **( Christian, 1986 )** **comb. n.**

*Onychiurus (Oligaphorura) hackeri* Christian, 1986 : 177.

*Type material*

Paratypes: 1 ♂ and 1 ♀, “Kranichberghöhle (2871/11), Gloggnitz, Niederösterreich SH=630 m, leg. E. Christian. 3.5.92”.19.X.1980. Type repository: NHMW.

*Redescription*

Body length 0.80–1.2 mm. Shape of body cylindrical. Colour white. Granulation homogenous, with coarse granules around all dorsal pseudocelli.

Antennae almost as long as head. Antennal segment I with 8 chaetae, antennal segment II with 13–14 chaetae. Sensory organ of antennal segment III consisting of 5 chaetae, 5 papillae, 2 smooth sensory rods, 2 rather granulated sense clubs, ventrolateral sensillum present. Antennal segment IV with subapical organite and microsensillum.

Postantennal organ vesicle with four (5) lobes, housed in small cuticular depression, and 1.4 as long as nearest pseudocellus. Labral formula of chaetae: 4/3, 4, 2. Maxillary palp simple with 2 sublobal hairs. Labial type ABC.

Pseudocellar formula dorsally: 32/022/33343, ventrally: 2/000/0000. Parapseudocelli not seen. All subcoxa 1 with 1 pseudocellus.

Dorsal chaetotaxy symmetrical, chaetae relatively long, well differentiated into macrochaetae and microchaetae. Sensory chaetae s on body weakly differentiated. Thoracic tergum I with 6+6 chaetae. Thoracic tergum II with lateral microsensilla, tergum III without microsensilla. Abdominal tergum VI with medial chaetae a0 and p0. Subcoxae 1 of I–III legs with 2, 3, 3 chaetae respectively. Thoracic sterna I–III with 0+0, 1+1, 1+1 chaetae respectively. Ventral tube with 7+7 chaetae, and 2+2 chaetae at base.

Furcal rudiment: a fine granulated area and three rows of chaetae behind its posterior edge. Row ma with 4 chaetae, row mm with only 2 external chaetae, and row mp with 4 chaetae.

Tibiotarsi I–III with 17, 17, 16 chaetae respectively. Distal tibiotarsal whorl with 9 chaetae. Claw without denticle. Empodial appendage with large basal lamella and 0.65 times of inner edge of claw.

Anal spines 0.33 times of inner claw edge and 5.4 times as long as their basal diameter.

*Distribution*

Austria: Lower Austria, Kranichberg cave.

*Dimorphaphorura inya* **Weiner & Kaprus’, sp. n.** ( Figures 19–26 )

*Type material*

Holotype ♀ on slide, Russia: Central Altai, vicinity Inya village, boulder ridge near Katun river, under a barberry, soil, 14.IX.1988, leg. S.K. Stebaeva & W.M. Weiner. Paratypes:7 ♀♀ on slides, the same data as holotype. Type repository: ISEA – holotype, paratypes: 2 ♀♀ and juv. ♂, MNH – paratype: 1 ♀, MSPU – paratype: 1 ♀, SNHM – paratypes: 4 ♀♀.

*Description*

**Figure 19-28.**
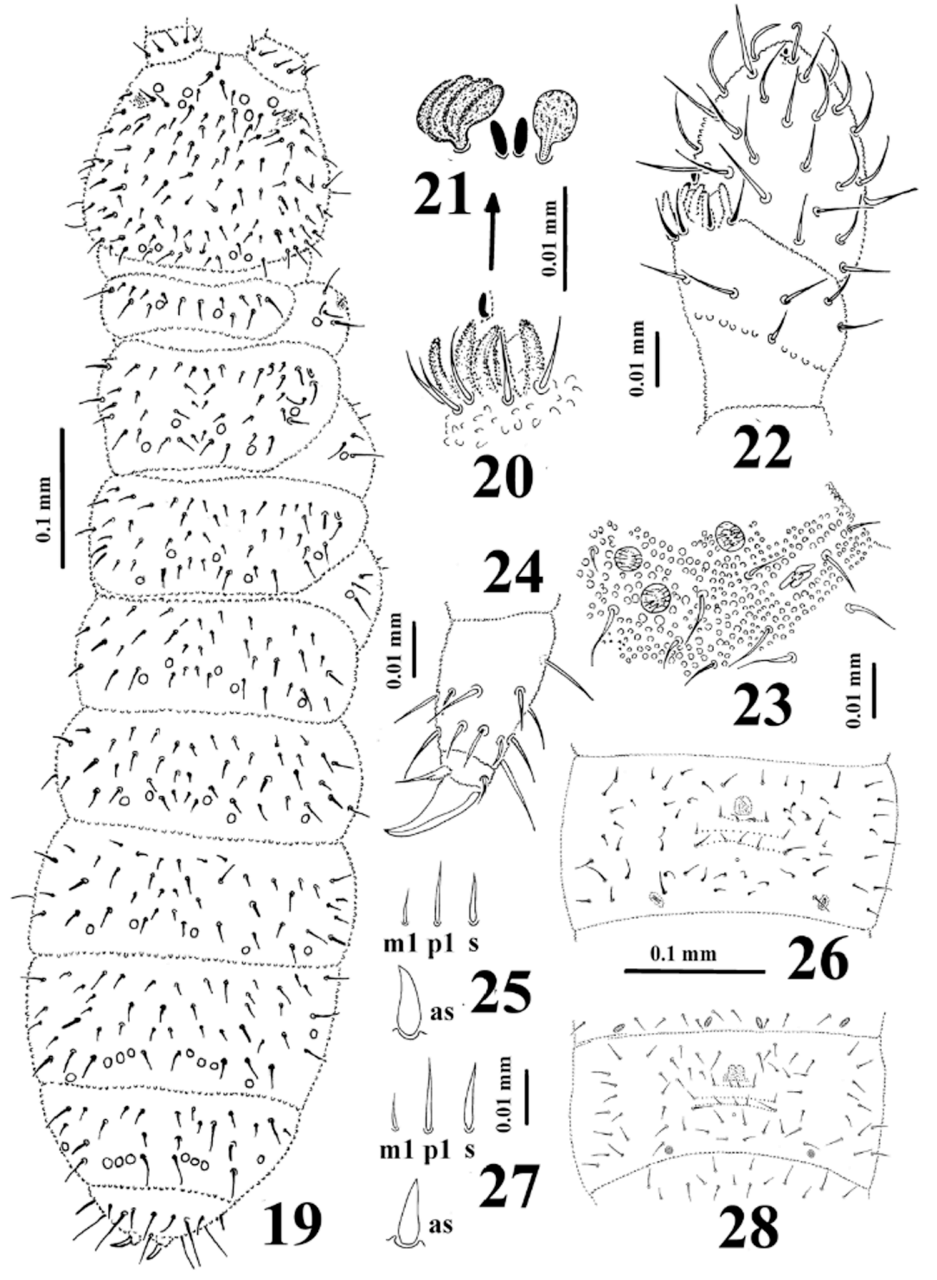
*Dimorphaphorura inyae* **sp. n., 27-28.** *Dimorphaphorura pseudoinyae* **sp. n.** : **19** , dorsal body chaetotaxy; **20** , papillae and guard chaetae of antennal III sense organ; **21** , sensory clubs and sensory rods; **22** , antennal segments III and IV with antennal III sense organ; **23** , postantennal organ and pseudocelli at base of antenna; **24** , tibiotarsal chaetotaxy and claw of leg III; **25** , **27** , ordinary chaetae (m1, p1) and chaeta s on abdominal tergum V and anal spine (as); **26** , **28** , chaetotaxy of abdominal sternum IV. High quality figures are available online.

Holotype length 0.79 mm, paratypes 0.70– 0.84 mm. Shape of body cylindrical ( Figure 19 ). Colour in alcohol white. Granulation homogenous, with coarse granules around all dorsal pseudocelli. Usually 11 grains around each pseudocellus.

Antennae almost as long as head. Antennal segment I with 8 chaetae, antennal segment II with 14 chaetae. Sensory organ of antennal segment III consisting of 5 chaetae, 5 papillae, 2 smooth sensory rods, and 2 ribbed sense clubs (internal clubs straight and globular, external ones bigger, ribbed, and bent), ventrolateral sensillum present ( Figures 20–22 ). Antennal segment IV with subapical organite and microsensillum ( Figure 22 ).

Postantennal organ vesicle with three lobes, housed in small cuticular depression, 1.6–1.7 as long as nearest pseudocellus ( Figure 23 ). Labral formula of chaetae: 4/3, 4, 2. Maxillary palp simple with 2 sublobal hairs. Labial type AC.

Pseudocellar formula dorsally: 32/133/33354; ventrally: 2/000/0000. Parapseudocellar (psx) formula ventrally: 1/000/212101 (on anal valves unpaired psx). Subcoxae 1 with 1 pseudocellus and 1 parapseudocellus (psx) ( Figures 19 , 26 ).

Dorsal chaetotaxy symmetrical, as in Figure 19 . Chaetae relatively short, well differentiated into macrochaetae and microchaetae. Sensory chaetae s on body distinct, distributed per half tergum as 2/011/222111. Thoracic terga II–III with lateral microsensilla. Abdominal tergum IV with or without medial chaeta m0. Abdominal tergum VI with medial chaeta a0 and p0. Shape and length of some ordinary chaetae, sensory chaeta s (on abdominal tergum V) and anal spines as in Figure 25 . Subcoxa 1 of legs I–III with 4, 5, 5 chaetae respectively. Chaetotaxy of abdominal sternum IV as in Figure 26 . Thoracic sterna I– III with 0+0, 1+1, 1+1 chaetae respectively. Ventral tube with 7-8+7-8 chaetae, and 1-3+1-3 chaetae at base.

Furcal rudiment: fine granulated area with three rows of chaetae behind its posterior edge. Row ma with 4 chaetae, row mm with 2 external chaetae (mm3), and row mp with 4–5 chaetae. ( Figure 26 ).

Tibiotarsi I–III with 20, 20, 19 chaetae respectively. Distal tibiotarsal whorl with 11 chaetae. Claw without denticle. Empodial appendage with distinct basal lamella and 0.67 of inner edge of claw ( Figure 24 ).

Anal spines 0.79 times as long as inner edge of claw and 3.17 times as long as their basal diameter.

*Remarks*

The new species, together with *D. alnus, D. sibirica* , and *D. pseudoinyae* , belongs to the group of species with 11 chaetae in the tibiotarsal distal whorl ( Table 1 ). They also have microsensilla on thoracic terga II and III, the same type of labial palp (AC), and the same number of dorsal pseudocelli on the head, thorax, and abdomen I–III (32/133/333). *D. inyae* and *D. pseudoinaye* present the same number of pseudocelli on abdominal terga IV and V (5 and 4), while the others have 4 and 3 pseudocelli. *D. sibirica* and *D. inyae* do not have pseudocelli on abdominal sternum IV, unlike to *D. alnus* and *D. pseudoinyae* , which carry 1+1 pseudocelli. The most similar species, *D. inyae* and *D. pseudoinyae,* live in different environmental conditions. *D. inyae* was found on the boulder ridge of Katun river while *D. pseudoinyae* in Siberian steppe.

*Etymology*

The name of the new species refers to the type locality in Inya village.

*Distribution*

Russia: Central Altai.

*Dimorphaphorura irinae* ( Thibaud & Taraschuk, 1997 ) comb. n.

*Micraphorura irinae* Thibaud & Taraschuk, 1997 : 113.

*Type material*

Holotype ♀ and paratypes: 2 ♀♀, Ukraine, Mykolaiv Region, in the neighborhood of Voznesens’k (Buz’ke), pine forest, sandy soil, 8.X.1995, J.-M. Thibaud). Type repository: MNHN.

*Other material*

Ukraine: Dnipropetrovs’k Region, Novomoskovs’k District, *Robinia pseudoacacia* , soil and litter, 23.VI.1984, 1 ♀, leg. N.O. Kuznetsova; Republic of Moldova: Vîşcăuţi, in the moss of the calcareous soil, 13.II. 2009, 1 ♀, 1 ♂, 3 juveniles, leg. G. Buşmachiu.

*Redescription*

Body length 0.55–0.61 mm. Body shape cylindrical. Colour white. Granulation homogenous, with coarse granules around all dorsal pseudocelli. Usually 9–10 grains around each pseudocellus.

Antennae approximately as long as head. Antennal segment I with 8 chaetae, antennal segment II with 13–14 chaetae. Sensory organ of antennal segment III consisting of 5 chaetae, 5 papillae, 2 smooth sensory rods, 2 weakly granulated sense clubs (internal clubs straight and globular, external ones bigger, ribbed and bent), ventrolateral sensillum present. Antennal segment IV with subapical organite and microsensillum.

Postantennal organ vesicle with 4 (3) lobes, located in small cuticular depression, 1.2 as long as nearest pseudocellus. Labral formula: 4/3, 4, 2. Maxillary outer lobe with simple palp and 2 sublobal hairs. Labial type ABC.

Pseudocellar formula dorsally: 32/133/33343, ventrally: 2/000/0001. Parapseudocellar (psx) formula ventrally: ?/000/111101 (on anal valves unpaired psx). All subcoxa 1 with 1 pseudocellus and 1 parapseudocellus.

Dorsal chaetotaxy symmetrical, chaetae relatively short, poorly differentiated into macro- and microchaetae. Body sensory chaetae s distinct, distributed per half tergum as 2/011/22211. Thoracic tergum I with 6-7+6- 7(8) chaetae. Thoracic terga II–III with lateral microsensilla. Abdominal tergum IV with medial chaeta m0. Abdominal tergum VI with medial chaeta a0 and p0. Subcoxa 1 of legs I– III with 3(4), 3(4), 3(4) chaetae respectively. Thoracic sterna I–III with 0+0, 1+1, 1+1 chaetae respectively. Ventral tube with 7+7 distal and 2+2 basal chaetae.

Furcal rudiment: fine granulated area and three rows of chaetae behind its posterior edge. Row ma with 4 chaetae, rows mm and mp with 2 and 4 chaetae, respectively.

Tibiotarsi I–III with 18, 18, 17 chaetae respectively. Distal tibiotarsal whorl with 9 chaetae. Claw without denticle. Empodial appendage 0.55 of claw inner edge and without distinct basal lamella.

Anal spines 0.70 times as inner edge of claw and 2.2 times as long as their basal diameter.

*Distribution*

Ukraine: Mykolaiv and Dnipropetrovs’k Regions, Moldova: Vîşcăuţi.

*Dimorphaphorura melittae* **( Christian, 1993 )** **comb. n.**

*Onychiurus (Oligaphorura) melittae* Christian, 1993 : 163

*Type material*

Paratypes, 6 ♂♂, “Windröhre, Brandgegend, Puchenstuben, NÖ, SH 655 m, 3.V.1992”, E. Christian. Type repository: E. Christian’s collection.

*Redescription*

Body length 0.65–0.80 mm (examined paratypes: 0.67–0.71 mm). Body shape cylindrical. Colour white. Granulation homogenous, with coarse granules around all dorsal pseudocelli. Usually 9–10 grains around each pseudocellus.

Antennae approximately as long as head. Antennal segment I with 8 chaetae, antennal segment II with 13 chaetae. Sensory organ of antennal segment III consisting of 5 chaetae, 5 papillae, 2 smooth sensory rods, 2 weakly granulated sense clubs (internal clubs straight and globular, external ones bigger, ribbed and bent), ventrolateral sensillum present. Antennal segment IV with subapical organite and microsensillum.

Postantennal organ vesicle 1.6–1.8 as nearest pseudocellus (in paratypes), with 3 (4) lobes, and located in small cuticular depression. Labral chaetotaxy not seen. Labial type ABC. Pseudocellar formula dorsally: 32/022/22243, ventrally: 2/000/0000. Parapseudocelli not seen. All subcoxa 1 with 1 pseudocellus.

Dorsal chaetotaxy symmetrical, chaetae relatively short, poorly differentiated into macrochaetae and microchaetae. Sensory chaetae s on body weakly differentiated. Thoracic tergum I with 4-5+5 chaetae. Thoracic tergum II with lateral microsensilla, tergum III with or without microsensilla (according to Christian (1993) , only 15% of individuals carry microsensilla, sometimes asymmetrically). Abdominal tergum IV with medial chaeta m0. Abdominal tergum VI with medial chaeta a0 and p0. Subcoxa 1 of legs I–III with 2, 3, 3 chaetae respectively. Thoracic sterna I–III with 0+0, 1+1, 1+1 chaetae respectively. Ventral tube with 5-7+5-7 chaetae, and 1+1 chaetae at base.

Furcal rudiment: fine granulated area and three rows of chaetae behind its posterior edge. Row ma with 4 chaetae, rows mm and mp with 2 and 4 chaetae, respectively.

Tibiotarsi I–III with 18, 18, 17 chaetae respectively. Distal tibiotarsal whorl with 9 chaetae. Claw without denticle. Empodial appendage shorter than claw (about 0.72 of inner edge of claw), with small basal lamella.

Anal spines 0.6–0.71 of length of claw inner edge and 3.4–4.0 times as long as their basal diameter.

*Distribution*

Austria: Lower Austria.

*Dimorphaphorura olenae* **Weiner & Kaprus’, sp. n.** ( Figures 29–36 )

*Type material*

Holotype ♂ on slide: Ukraine, Donets’k district, Kamiani Mohyly Reserve, steppe plant community, soil, 23.X.1996, leg. O. Starostenko. Paratypes: 1 ♂ and 1 ♀♀ on slides, the same data as holotype. Type repository: SNHM – holotype and paratype: 1 ♀; ISEA – paratype: 1 ♂.

*Other material*

Ukraine: Dnipropetrovs’k district, Novomoskovs’k region, Kapitanovs’kyi bajrak, steppe plant community, soil in ravine, 25.VI.1985, 1 ♀ I.P. Vtorov.

*Description*

**Figure 29-36.**
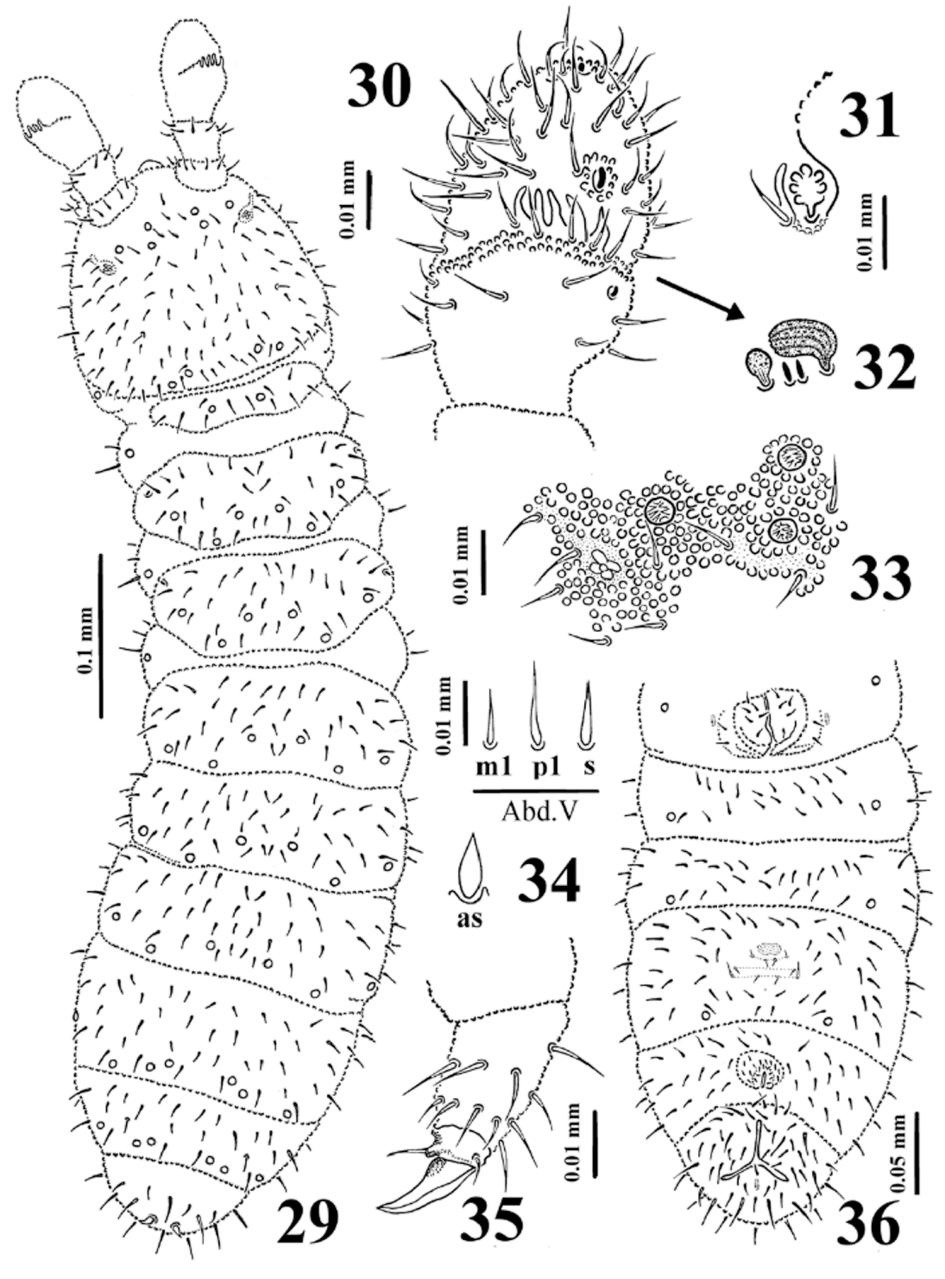
*Dimorphaphorura olenae* **sp. n.** : **29** , dorsal body chaetotaxy; **30** , antennal segments III and IV with antennal III sense organ; **31** , cross section of antennal III sense organ; **32** , sensory clubs and sensory rods; **33** , postantennal organ and pseudocelli at base of antenna; **34** , ordinary chaetae (m1, p1) and chaeta s on abdominal tergum V, and anal spine (as); **35** , tibiotarsal chaetotaxy and claw of leg III; **36** , chaetotaxy of abdominal sterna I-VI. High quality figures are available online.

Holotype length 0.60 mm, length of paratypes: 0.53–0.78 mm. Body shape cylindrical ( Figure 29 ). Colour in alcohol white. Granulation homogenous, with coarse granules around all dorsal pseudocelli. Usually 10 grains around each pseudocellus.

Antennae shorter than head. Antennal segment I with 8 chaetae, antennal segment II with 12–13 chaetae. Sensory organ of antennal segment III consisting of 5 chaetae, 5 papillae, 2 smooth sensory rods, 2 weakly granulated sense clubs (internal clubs straight and globular, external ones bigger, ribbed and bent) ( Figures 30–32 ), ventrolateral sensillum present. Antennal segment IV with subapical organite and microsensillum ( Figure 30 ).

Postantennal organ vesicle as long as nearest pseudocellus, with 3 lobes, and housed in small cuticular depression ( Figure 33 ). Labral formula of chaetae: 4/3, 4, 2. Maxillary outer lobe with simple palp and 2 sublobal hairs. Labial type ABC.

Pseudocellar formula dorsally: 32/133/33343, ventrally: 2/000/1111 ( Figures 28 , 35 ). Parapseudocellar formula ventrally: ?/000/100001 (on anal valves unpaired psx) ( Figure 36 ). Subcoxa 1 of legs I–III with 1, 2, 2 pseudocelli and 1, 1, 1 parapseudocellus respectively.

Dorsal chaetotaxy symmetrical, as in Figure 29 . Chaetae relatively short, poorly differentiated into macro- and microchaetae. Sensory chaetae s on body poorly differentiated also. Thoracic terga II–III with lateral microsensilla. Abdominal tergum IV with or wothout medial chaeta m0. Abdominal tergum VI with medial chaeta a0 and p0. Shape and length of some ordinary chaetae, sensory chaeta s (on abdominal tergum V) and anal spines as in Figure 34 . Subcoxa 1 of legs I–III with 3, 4(3), 4(3) chaetae respectively. Chaetotaxy of abdominal sterna as in Figure 36 . Thoracic sterna I–III with 0+0, 1+1, 1(2)+1 chaetae respectively. Ventral tube with 5-6+5-6 distal and 2+2 basal chaetae.

Furcal rudiment: fine granulated area and three rows of chaetae behind its posterior edge. Row ma with 4 chaetae, rows mm and mp with 2 and 4 chaetae, respectively ( Figure 36 ).

Tibiotarsi I–III with 18, 18, 17 chaetae respectively. Distal tibiotarsal whorl with 9 chaetae. Claw without denticle. Empodial appendage shorter than claw (0.55 of inner edge of claw), without basal lamella ( Figure 35 ).

Anal spines 0.50 of length of inner edge of claw and 2.0 times as long as their basal diameter.

*Remarks*

See remarks in *D. sophyae***sp. n.** and Table 1 .

*Etymology*

The new species is dedicated to Olena Starostenko, who collected the material of the species.

*Distribution*

Ukraine: Donets’k and Dnipropetrovs’k districts.

*Dimorphaphorura pseudoinya* **Weiner & Kaprus’, sp. n.** ( Figures 27 , 28 )

*Type material*

Holotype ♀ on slide: Russia, Krasnoyarsk Territory, ca 5–7 km S of Nazarovo, upper part of natural katena, herb-grass steppified meadow, meadow-chernozem soil, 5 cm depth, in decaying roots of *Melilotus albus,* 20.VIII.1988, leg. S.K. Stebaeva. Paratypes, 20 ♀♀, 5 ♂♂ on slides: the same locality as the holotype. Type repository: SNHM – holotype and paratypes: 11 ♀♀ and 3 ♂♂, MSPU – paratypes: 3 ♀♀ and 1 ♂, MNH – paratypes: 3 ♀♀, ISEA – paratypes: 3 ♀♀ and 1 ♂.

*Description*

Holotype length 0.66 mm, length of paratypes: 0.62–0.82 mm. Body shape cylindrical. Colour in alcohol white. Granulation homogenous, with coarse granules around all dorsal pseudocelli. Usually 11 grains around each pseudocellus.

Antennae almost as long as head. Antennal segment I with 8 chaetae, antennal segment II with 14 chaetae. Sensory organ of antennal segment III consisting of 5 chaetae, 5 papillae, 2 smooth sensory rods, 2 weakly granulated sense clubs (internal clubs straight and globular, external ones bigger, ribbed and bent), ventrolateral sensillum present (as in *D. inya***sp. n.** , Figures 20–22 ). Antennal segment IV with subapical organite and microsensillum.

Postantennal organ vesicle 1.6–1.7 times as long as nearest pseudocellus, with 3 lobes and located in a small cuticular depression . Labral formula: 4/3, 4, 2. Maxillary outer lobe with simple palp and 2 sublobal hairs. Labial type AC.

Pseudocellar formula dorsally: 32/133/33354, ventrally: 2/000/0001 ( Figure 28 ). Parapseudocellar (psx) formula ventrally: 1/000/212001 (on anal valves unpaired psx). All subcoxa 1 with 1 pseudocellus and 1 parapseudocellus.

Dorsal chaetotaxy symmetrical, as in *D. inya***sp. n.** ( Figure 19 ). Chaetae relatively short, well differentiated into macro- and microchaetae. Body sensory chaetae s distinct, distributed as 2/011/222111. Thoracic terga II–III with lateral microsensilla. Abdominal tergum IV with or without medial chaeta m0. Abdominal tergum VI with medial chaeta a0 and p0. Shape and length of some ordinary chaetae, sensory chaeta s (on abdominal segment V) and anal spines as in Figure 27 . Subcoxa 1 of legs I–III with 4, 5, 5 chaetae respectively. Chaetotaxy of abdominal sternum IV as in Figure 28 . Thoracic sterna I–III with 0+0, 1+1, 1+1 chaetae respectively. Ventral tube with 7-8+7-8 distal and 1-3+1-3 basal chaetae.

Furcal rudiment: fine granulated area with three rows of chaetae behind its posterior edge. Row ma with 4 chaetae, rows mm and mp with 2 and 4–5 chaetae respectively.

Tibiotarsi I–III with 20, 20, 19 chaetae respectively. Distal tibiotarsal whorl with 11 chaetae. Claw without denticle. Empodial appendage shorter than claw (0.67 of claw inner edge), with distinct basal lamella (as in *D. inya***sp. n.** , Figure 24 ).

Anal spines 0.72 of length of claw inner edge and 2.6 times as long as their basal diameter.

*Remarks*

See remarks in *D. inya***sp. n.** and Table 1 .

*Etymology*

The name of the new species is derived from the similar species *D. inya***sp. n.**

*Distribution*

Russia: Krasnoyarsk Territory.

*Dimorphaphorura pseudoraxensis* **( Nosek & Christian, 1983 ) comb. n.**

*Onychiurus (Oligaphorura) pseudoraxensis* , Nosek & Christian, 1983 : 397.

*Type material*

Holotype ♀ on slide: “Wandschluf (Kat. Nr. 1823/34) im Schöpftaler Wald bei Lunz am See, Niederösterreich. Koord, 47°50’/14°58’. Seehöhe: 900m, 6.V.1978”, leg. E. Christian. Type repository: MNH.

*Redescription*

Body length 1.1–1.15 mm. Body cylindrical. Colour white. Granulation homogenous, with coarse granules around all dorsal pseudocelli. Usually 12 grains around each pseudocellus. Antennae approximately as long as head.

Antennal segment I with 6 chaetae visible, antennal segment II with 12 chaetae. Sensory organ of antennal segment III consisting of 5 chaetae, 5 papillae, 2 smooth sensory rods, 2 sense clubs rather granulated (slightly visible), ventrolateral sensillum present. Antennal segment IV with subapical organite and microsensillum.

Postantennal organ vesicle 1.25 as long as nearest pseudocellus, with 3(4) lobes and housed in a small cuticular depression. Labral chaetotaxy not seen. Labial type ABC.

Pseudocellar formula dorsally: 32/133/33343 (/33344 in the original description), ventrally: 2/000/0000. Parapseudocelli not seen. All subcoxa 1 with 1 pseudocellus.

Chaetotaxy symmetrical, chaetae relatively short, poorly differentiated into macro- and microchaetae. Body sensory chaetae s weakly differentiated. Thoracic tergum II with lateral microsensilla, tergum III obstructed. Abdominal tergum VI with medial chaeta a0 and p0. Subcoxa 1 of legs I–III with 2, 3, 3 chaetae respectively. Ventral tube with 7+7 distal and 2+2 basal chaetae.

Furcal rudiment: fine granulated area and three rows of chaetae behind its posterior edge. Row ma with 4 chaetae, rows mm and mp with 2 and 4 chaetae respectively.

Distal tibiotarsal whorl with 9 chaetae (all chaetae on tibiotarsi not seen). Claw without denticle. Empodial appendage shorter than claw (0.82 of claw inner edge), with large basal lamella.

Anal spines as spiniform chaetae 5.3 times as long as their basal diameter.

*Distribution*

Austria: Lower Austria.

*Dimorphaphorura raxensis* **( Gisin, 1961 )** **comb. n.**

*Onychiurus raxensis* Gisin, 1961 : 336

*Type material*

Paratypes: 1 ♂and 1 ♀: “Raxalpe (Niederösterreich, Alpen), auf Schneewasser und an Holz halb unter Schnee, 4.iv.1927, leg. C. Börner“. Type repository: MNH.

*Additions to the original description*

Ventral tube with 6+6 distal and 2+2 basal chaetae. Furcal rudiment comprising a fine granulated area and three rows of chaetae behind its posterior edge. Row ma with 4 chaetae, rows mm and mp with 2 and 4 chaetae respectively. Claw without denticle. Empodial appendage shorter than claw (0.8 of claw inner edge), with distinct basal lamella.

Anal spines 0.5 times as long as inner edge of claw and 2.0 times as long as their basal diameter.

*Distribution*

Austria: Lower Austria.

*Dimorphaphorura sibirica* **Weiner & Kaprus’, sp. n.** ( Figures 37–44 )

*Type material*

Holotype ♀ on slide: Russia, Western Siberia, 25 km S of Novosibirsk, Akademgorodok, glade in *Betula verrucosa* forest, soil, 1.V.1993, leg. S.K. Stebaeva. Paratypes: 5 ♀♀, 7 ♂♂ and 39 juv. on slides: the same data as holotype. Type repository: ISEA – holotype and paratypes: 2 ♀♀ and 1 ♂, SNHM – paratypes: 4 ♀♀, 1 ♂ and 31 juv., MSPU – paratypes: 3 ♂♂, 1 ♀ and 8 juv.

*Other material*

Russia, northeastern Altai, vicinity of Lake Teletskoye, ca 25 km S of Iogach, locality “Obogo,” low-lying Picea obovata forest with *Hylocomium splendens* , *Calamagrostis langsdorffii* , *Veratrum lobelianum* , 500 m alt., soil, 10.IX.1988, 1 ♂ and ♀; ca 7 km N-E of Artybash vil., upper stony part of mountain, ca 500–600 m alt., *Pinus sibirica* - *Abies sibirica* forest with Bergenia crassifolia and firns, soil, 0–5 cm, 10.IX.1988, 1 ♀, leg. W.M. Weiner & S.K. Stebaeva; Krasnoyarsk Territory, close to Nazarovo, nonlevelled 3-year-old brown coal mine dump, soil, 21.VI.1989, 1 ♀; Kemerovo Region, Kuznetskii Alatau, 10 km N-W of Mezhdurechensk, ca. 500–600 m alt., *Abies sibirica-Populus tremula* forest, glade with tall herbaceous vegetation, soil, 25–30 cm, 30.VI.1982, 1♀, leg. S.K. Stebaeva; ca 130 km S-E of Novosibirsk, 11 km N of Mirnyi, Salairskii mountain ridge, ca 500 m alt., *Abies sibirica-Populus tremula* forest, microdepression with tall herbaceous vegetation, soil, 0–5 cm, 6.VI.1972, 1 juv., leg. S.K. Stebaeva.

*Description*

**Figure 37-44.**
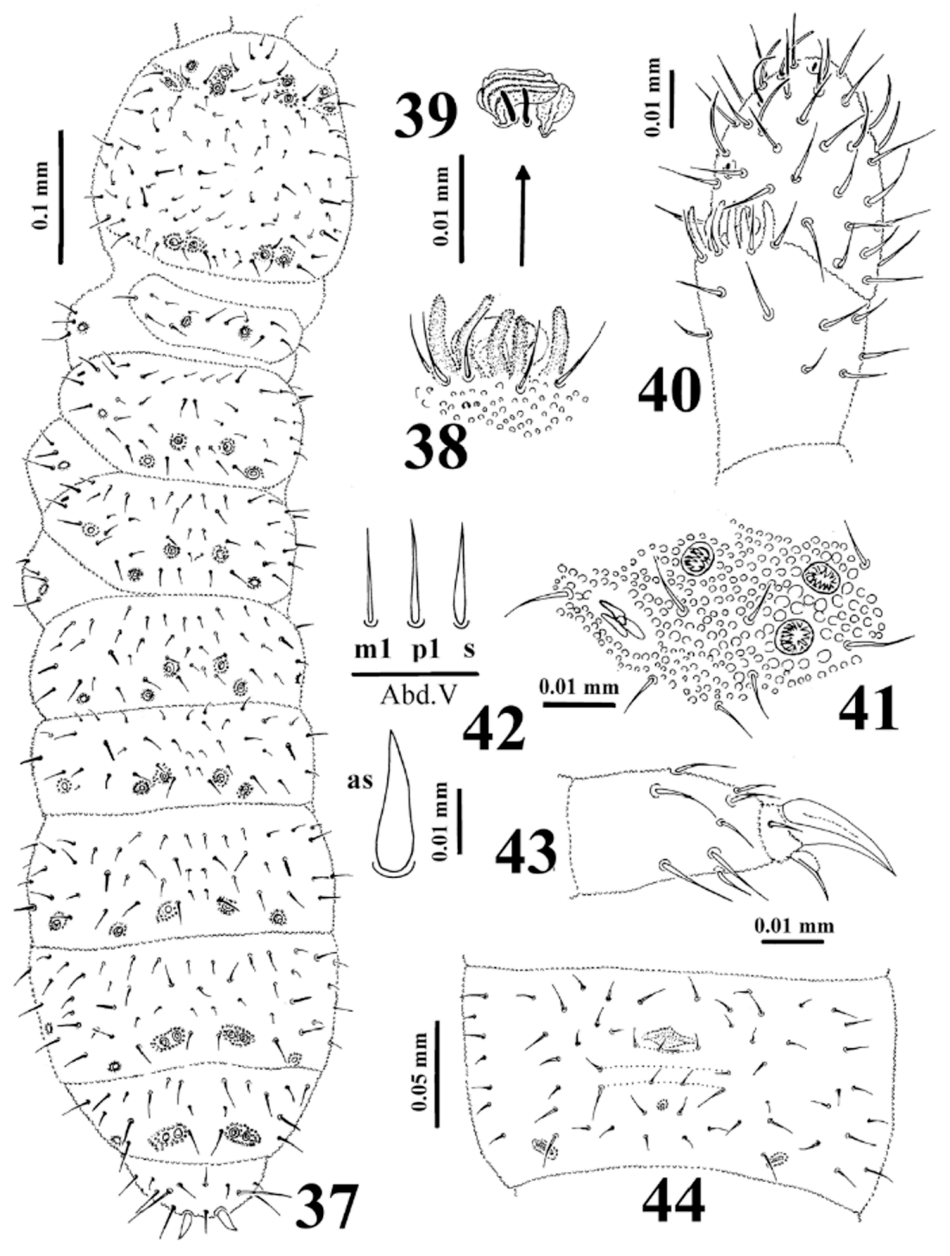
*Dimorphaphorura sibirica* **sp. n.** : **37** , dorsal body chaetotaxy; **38** , papillae and guard chaetae of antennal III sense organ; **39** , sensory clubs and sensory rods; **40** , antennal segments III and IV with antennal III sense organ; **41** , postantennal organ and pseudocelli at base of antenna; **42** , ordinary chaetae (m1, p1) and chaeta s on abdominal tergum V, and anal spine (as); **43** , tibiotarsal chaetotaxy and claw of leg III; **44** , chaetotaxy of abdominal sternum IV. High quality figures are available online.

Holotype length 0.92 mm, length of paratypes: 0.67–0.93 mm. Body cylindrical ( Figure 37 ). Colour in alcohol white. Granulation homogenous, with coarse granules around all dorsal pseudocelli. Usually 11 grains around each pseudocellus.

Antennae approximately as long as head. Antennal segment I with 8 chaetae, antennal segment II with 15 chaetae. Sensory organ of antennal segment III consisting of 5 chaetae, 5 papillae, 2 smooth sensory rods, 2 weakly granulated sense clubs (internal clubs straight and globular, external ones bigger, ribbed and bent), ventrolateral sensillum present ( Figures 38–40 ). Antennal segment IV with subapical organite and microsensillum ( Figure 40 ).

Postantennal organ vesicle 1.7–1.9 as long as nearest pseudocellus, with 3 lobes and located in a small cuticular depression ( Figure 41 ). Labral formula of chaetae: 4/3, 4, 2. Maxillary outer lobe with simple palp and 2 sublobal hairs. Labial type AC.

Pseudocellar formula dorsally: 32/133/33343 ( Figure 37 ), ventrally: 2/000/0000. Parapseudocellar formula ventrally: 1/000/212103 (each anal valve with parapseudocellum). Subcoxa 1 of legs I–III with 1, 1, 1 pseudocellus and 1, 2, 2 parapseudocelli respectively.

Dorsal chaetotaxy symmetrical, as in Figure 37 . Chaetae relatively short, well differentiated into macro- and microchaetae. Sensory chaetae s on body distinct, distributed as 2/011/22211. Thoracic terga II–III with lateral microsensilla. Abdominal tergum IV with medial chaeta m0. Abdominal tergum VI with medial chaeta a0 and p0. Shape and length of some ordinary chaetae, sensory chaetae s (on abdominal tergum V) and anal spine as in Figure 42 . Subcoxa 1 of legs I–III with 4, 5, 5 chaetae respectively. Chaetotaxy of abdominal sternum IV as in Figure 44 . Thoracic sterna I– III with 0+0, 1+1, 1+1 chaetae respectively. Ventral tube with 7-8+7-8 distal and 2-4+2-4 basal chaetae.

Furcal rudiment: small area with fine granulation and three rows of chaetae behind its posterior edge. Row ma with 4 chaetae, row mm with only 2 external chaetae, and row mp with 4–5 chaetae ( Figure 44 ).

Tibiotarsi I–III with 20, 20, 19 chaetae respectively. Distal tibiotarsal whorl with 11 chaetae. Claw without denticle. Empodial appendage shorter than claw (0.56 of inner edge of claw), with distinct basal lamella ( Figure 43 ).

Anal spines 0.95 times as inner edge of claw and 2.86 times as long as their basal diameter.

*Remarks*

Three other species *(D. alnus* , *D. inyae* , and *D. pseudoinyae)* have 11 chaetae in the distal tibiotarsal whorl, but the latter two species have 5+5 pseudocelli on abdominal tergum IV ( Table 1 ). *Dimorphaphorura sibirica* and *D. inyae* do not have sternal pseudocelli on abdomen IV, whereas *D. alnus* and *D. pseudoinyae* carry 1+1 pseudocelli.

*Etymology*

The name of the new species refers to the type locality that belongs to the studied region, Siberia.

*Distribution*

Russia: Siberia.

*Dimorphaphorura sophyae* **Weiner & Kaprus’, sp. n.** ( Figures 45–52 )

*Type material*

Holotype, ♀ on slide: Russia, Central Altai, before elevation to Seminsky Mt. pass, 1300 m alt., wet valley with *Abies sibirica* forest, soil, 16.IX.1988, leg. W.M.W. Weiner & S.K. Stebaeva. Paratypes: 4 ♀♀ and 5 ♂♂ on slides: the same data as holotype. Type repository: ISEA – holotype and paratypes: 2 ♂♂ and 1 ♀, MNH – paratypes: 1 ♂ and 1 ♀, SNHM – paratypes: 2 ♂♂ and 2 ♀♀.

*Other material*

Russia: Central Altai, Seminsky Mt. pass, 1500 m alt., *Pinus sibirica* forest, 16.IX.1988, 2 ♀♀ and 2 ♂♂, leg. W.M. Weiner & S.K. Stebaeva; N-E Altai, Teletskoye Lake, Altai Reserve, 15 km from Artybash vil., middle part of slope, *Larix sibirica* , litter and soil, 11.IX.1988, 3 ♀♀, leg. W.M. Weiner & S.K. Stebaeva.

*Description*

**Figure 45-52.**
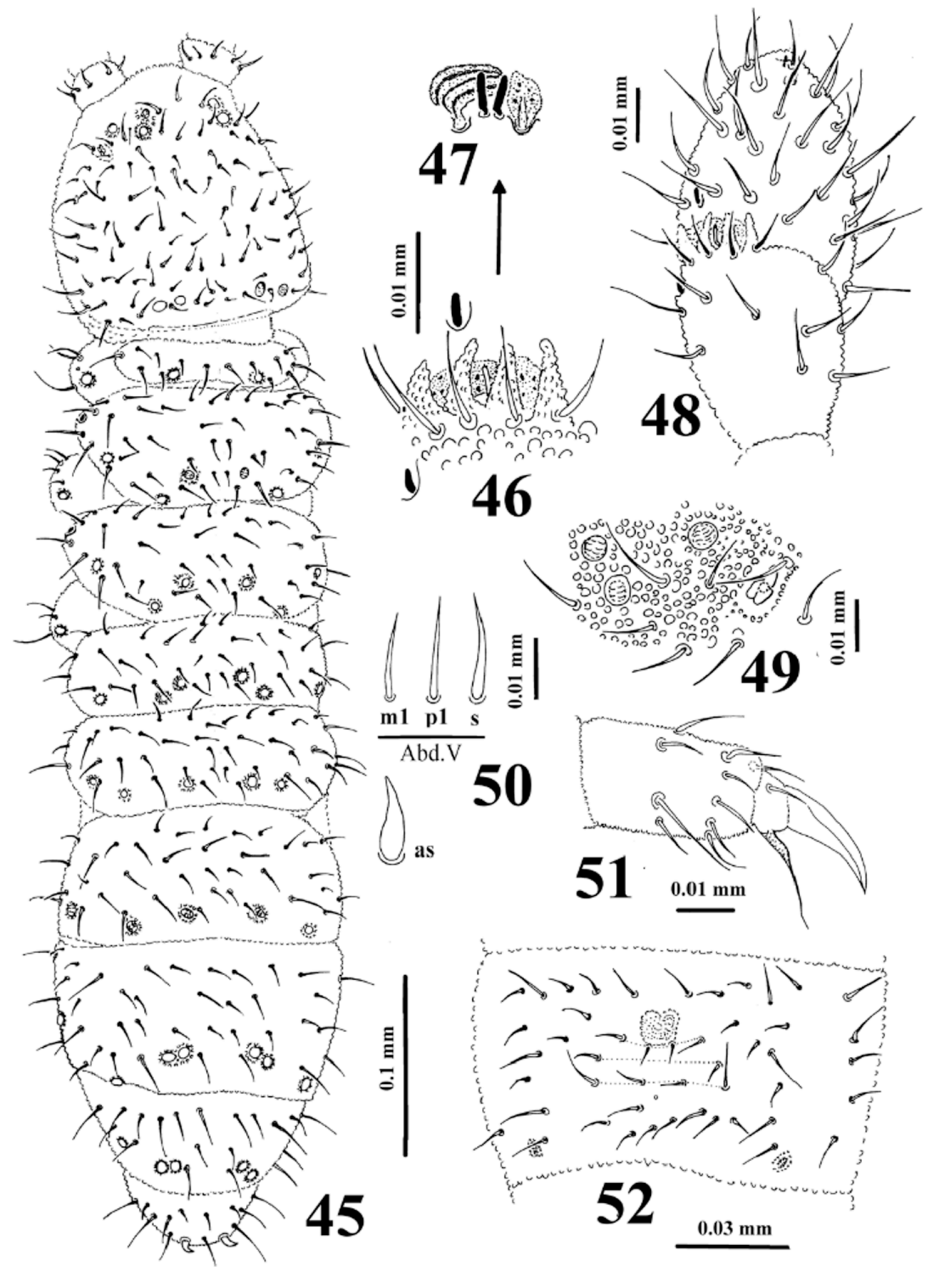
*Dimorphaphorura sophyae* **sp. n.** : **45** , dorsal body chaetotaxy; **46** , papillae and guard chaetae of antennal III sense organ; **47** , sensory clubs and sensory rods; **48** , antennal segments III and IV with antennal III sense organ; **49** , postantennal organ and pseudocelli at base of antenna; **50** , ordinary chaetae (m1, p1) and chaeta s on abdominal tergum V and anal spine (as); **51** , tibiotarsal chaetotaxy and claw of leg III; **52** , chaetotaxy of abdominal sternum IV. High quality figures are available online.

Holotype length 0.64 mm, length of paratypes 0.60–0.70 mm. Body shape cylindrical ( Figure 45 ). Colour in alcohol white. Granulation homogenous, with coarse granules around all dorsal pseudocelli. Usually 12 grains around each pseudocellus.

Antennae almost as long as head . Antennal segment I with 8 chaetae, antennal segment II with 14 chaetae. Sensory organ of antennal segment III consisting of 4 papillae, 5 chaetae, 2 smooth sensory rods, 2 weakly granulated sense clubs (internal clubs straight and globular, external ones bigger, ribbed and bent), ventrolateral sensillum present ( Figures 46– 48 ). Antennal segment IV with subapical organite and microsensillum ( Figure 48 ).

Postantennal organ vesicle 1.5 times as long as nearest pseudocellus, with 3 lobes and located in a small cuticular depression ( Figure 49 ). Labral chaetotaxy as 4/3, 4, 2. Maxillary outer lobe with simple palp and 2 sublobal hairs. Labial type AC.

Pseudocellar formula dorsally: 32/133/33343 ( Figure 45 ), ventrally: 2/000/0000. Parapseudocellar formula ventrally: 1/000/111103 (each anal valve with parapseudocellus). All subcoxa 1 with 1 pseudocellus and 1 parapseudocellus.

Dorsal chaetotaxy symmetrical, as in Figure 45 . Chaetae relatively short, well differentiated into macrochaetae and microchaetae. Body chaetae s distinct, distributed as 2/011/22211. Thoracic terga II–III with lateral microsensilla. Abdominal tergum IV with medial chaeta m0. Abdominal tergum VI with medial chaeta a0 and p0. Shape and length of some ordinary chaetae, sensory chaeta s (on abdominal tergum V) and anal spines as in Figure 50 . Subcoxa 1 of legs I–III with 4, 5(4), 5(4) chaetae respectively. Chaetotaxy of abdominal sternum IV as in Figure 52 . Thoracic sterna I– III with 0+0, 1+1, 1+1 chaetae respectively. Ventral tube with 6-7+6-7 distal, and 1-2+1-2 basal chaetae.

Furcal rudiment: fine granulated area and three rows of chaetae behind its posterior edge. Row ma with 4 chaetae, rows mm and mp with 2 and 4 chaetae respectively ( Figure 52 ).

Tibiotarsi I–III with 18, 18, 17 chaetae respectively. Distal tibiotarsal whorl with 9 chaetae. Claw without denticle. Empodial appendage equal or longer than claw (1.0–1.1 of claw inner edge), with small basal lamella ( Figure 51 ).

Anal spines 0.77 times as long as inner edge of claw and 3.0 times as long as their basal diameter.

*Remarks*

Between the species of the genus *Dimorphaphorura* , only *D. sophyae***sp. n.** has four papillae in the sensory organ of antenna III and empodial appendage equal or longer than claw ( Table 1 ). Based on the pseudocellar formula and the number of chaetae in the distal tibiotarsal whorl (9), the new species is most similar to *D. pseuraxensis* . Besides the number of papillae in the sensory III organ, both species differ in the type of labial palp (AC in *D. sophyae* and ABC in *D. pseudoraxensis* ) and in the length of empodial appendage (empodium equal or longer than claw in *D. sophyae* and shorter than claw in *D. pseudoraxensis* ). Two other species, *D. irinae* and *D. olenae* , have the same dorsal pseudocellar formula and number of distal tibiotarsal chaetae (9), but they differ in the pseudocellar formula of abdominal sterna I– IV (0001 in *D. irinae* , 1111 in *D. olenae* , absent in *D. sophyae* and *D. pseudoraxensis* ) and also in the length of empodial appendage (empodium shorter than claw in *D. irinae* and *D. olenae* ).

*Etymology*

The new species is dedicated to Dr. Sophya K. Stebaeva, a well-known researcher of Siberian collembolan fauna and our friend.

*Distribution*

Russia: Central Altai.

*Dimorphaphorura steposa* **( Kaprus’, Weiner & Pomorski, 2002 ) comb. n.**

*Micraphorura steposa* Kaprus’, Weiner & Pomorski, 2002 : 353.

*Type material*

Holotype ♀ and paratype 1 ♀ on slides: Ukraime, Donets’ka district, Khomutovskiy Steppe Reservation, steppe vegetation, mowed, soil, 4.V.1996, leg. O. Starostenko. Type repository: SNHM.

*Remarks*

Labral formula of chaetae: 4/3, 4, 2. Maxillary outer lobe with simple palp and 2 sublobal hairs. Labial type AC. Thoracic tergum I with 5-6+5-6(7) chaetae. Subcoxa 1 of legs I–III, with 2(3), 3(4), 3 chaetae respectively. Furcal rudiment consisting of a fine granulated area and three rows of chaetae behind its posterior edge. Row ma with 4 chaetae, rows mm and mp with 2 and 4 chaetae respectively.

*Distribution*

Ukraine: Donets’ka district.

*Dimorphaphorura sanjiangensis* **Sun & Wu, 2012** : 106

*Distribution*

China, Heilongjiang Province, Honghe Farm.

*Dimorphaphorura jingyueensis* **Sun & Wu, 2012** : 46

*Distribution*

China, Jilin Province, Jingyuetan National Forest Park.

*Dimorphaphorura stojkoae* ( Shvejonkova & Potapov, 2011 ) **comb. n.**

*Micraphorura stojkoae* Shvejonkova & Potapov, 2011 : 353

*Distribution*

Russia (European part), Middle Volga River Basin.

*Remarks*

The species was described by Shvejonkova and Potapov (2011) in the genus *Micraphorura* . However, it possess characters allowing the transfer of this species to the genus *Dimorphaphorura* . The arragment of the furcal area is typical to the latter genus: without pocket and dental setulae, with only external (1+1) mm chaetae. The species also posses also some distinct (vs. indistinct in species of *Micraphorura* ) chaetae s on the body and a lower number (7) of chaetae in tibiotarsal distal row.

### Key to species of world *Dimorphaphorura*

1. Anal spines absent, antennal III sense organ with 5 papillae, tibiotarsal distal whorl with 7 chaetae, pseudocellar formula dorsally: 32/133/33343, ventrally: 2/000/00000, empodial appendage ¾ long as claw inner edge … …………. ****D. stojkoae**** (Russia, European part) – Anal spines or spiniform chaetae present, antennal III sense organ with 4 or 5 papillae, tibiotarsal distal whorl with 11 or less chaetae.. ………………………………………………2

2. Antennal III sense organ with 4 papillae, tibiotarsal distal whorl with 9 chaetae, pseudocellar formula dorsally: 32/133/33343; ventrally: 2/000/00000, empodial appendage equal or longer than claw inner edge……...…. ………….. *D. sophyae***sp. n** . (Russia, Siberia) – Antennal III sense organ with 5 papillae, other characters variable……………………..3

3. Tibiotarsal distal whorl with 11 chaetae, labial type AC or A………………………….4 – Tibiotarsal distal whorl with 9 or less chaetae, labial type AC or ABC………………….9

4. Empodial appendage as long as inner edge of claw, male ventral organ present on ventral tube and on Abd. II–IV, pseudocellar formula dorsally: 32/133/33353, ventrally: 2/000/00000………………………………… …………. *D. jingyueensis***(** China, Jilin Prov. **)** – Empodial appendage as 0.50–0.75 of inner edge of claw, male ventral organ absent…….5

5. Abdominal terga IV–V with 4, 3 pseudocelli, respectively……………………………….6 – Abdominal terga IV–V with 5, 4 pseudocelli, respectively……………………………….8

6. Thoracic terga I–III with 0, 3, 3 pseudocelli, respectively, labial type A, abdominal tergum IV with a0 and m0……………………. …...... ****D. sanjiangensis**** (China, Heilonggjiang Prov.) – Thoracic terga I–III with 1, 3, 3 pseudocelli, respectively, labial type AC, abdominal tergum IV only with m0………………………..7

7. Abdominal sternum IV with 1+1 pseudocelli, subcoxa 1 of legs I–III with 3, 3, 3 chaetae, respectively………. ***D. alnus*** (Russia, Siberia) – Abdominal sternum IV without pseudocelli, subcoxa 1 of legs I–III with 4, 5, 5 chaetae, respectively………. ***D. sibirica*** sp. n. (Russia, Siberia)

8. Abdominal sternum IV with 1+1 pseudocelli, anal spines 2.6 times as long as their basal diameter………. ***D. pseudoinya*sp. n.** (Russia, Siberia) – Abdominal sternum IV without pseudocelli, anal spines 3.2 times as long as their basal diameter……….. ***D. inya*sp. n.** (Russia, Siberia)

9. Tibiotarsal distal whorl with 9 chaetae….10 – Tibiotarsal distal whorl with less than 9 chaetae……………………………………..18

10. Thoracic tergum I with 1+1 pseudocelli, microsensilla ms on thoracic tergum III present…………………………………………11 – Thoracic tergum I without pseudocelli, microsensilla ms on thoracic tergum III present or absent III………………………………...15

11. Abdominal sterna I–IV without pseudocelli……………………………………………12 – At least 1+1 pseudocelli on abdominal sternum IV……………………………………..14

12. Subcoxae 1 of legs I–III with 2, 3, 3 chaetae, respectively, anal spines as spiniform chaetae (5.3 times as long as their basal diameter), pseudocellar formula dorsally: 32/133/33343….. *D. pseudoraxensis* (Austria) – Subcoxae 1 of legs I–III with more chaetae, anal spines different (less than 2.5 times as long as their basal diameter)……………….13

13. Subcoxae 1 of legs I–III with 3, 3, 3 chaetae, respectively, dorsal side of body with homogenous granulation… ***D. caucasica*sp. n.** (Russia, North Caucasus) – Subcoxae 1 of legs I–III with 4, 4, 5 chaetae, respectively, abdominal tergum VI and head with coarse granulation………………… ……………………... ****D. differens**** (Austria)

14. Subcoxae 1 of legs I–III with 1, 1, 1 pseudocelli, respectively, pseudocellar formula of abdominal sterna I–IV as 0001………………. *……………….…* . ***D. irinae*** (Ukraine, Moldova) – Subcoxae 1 of legs I–III with 1, 2, 2 pseudocelli, respectively, pseudocellar formula of abdominal sterna I–IV as 1111………………. …………………… *D. olenae***sp. n** . (Ukraine)

15. Thoracic terga I–III with 0, 3, 3 pseudocelli, respectively, microsensilla on thoracic tergum III present……... ****D. raxensis**** (Austria) – Thoracic terga I–III with 0, 2, 2 pseudocelli, respectively…………………………….......16

16. Abdominal terga I, II with 3, 3 pseudocelli, respectively (pseudocellar formula dorsally: 2/022/33343, thoracic tergum III without icrosensilla…………… ****D. hackeri**** (Austria) Abdominal terga I, II with 2, 2 pseudocelli, respectively………………………………...17

17. Dorsal pseudocellar formula: 2/022/22243, abdominal sternum IV without pseudocelli……………... ****D. melittae**** (Austria) Dorsal pseudocellar formula: 32/022/22343, abdominal sternum IV with 1+1 pseudocelli………………………... ****D. eremia**** (Ukraine)

18. Tibiotarsal distal whorl with 6 chaetae, microsensilla on thoracic tergum III absent….. *i>……………………………..* . ***D. daii*** (Ukraine) Tibiotarsal distal whorl with 5 chaetae, microsensilla on thoracic tergum III present…19

19. Thoracic terga I–III with 0, 3, 3 pseudocelli, respectively, tibiotarsi I–III with 13, 13, 2 chaetae, respectively……………………… …………………... *D. chatyrdagi* (Ukraine) Thoracic terga I–III with 0, 2, 2 pseudocelli, respectively, tibiotarsi I–III with 12, 12, 11 chaetae, respectively…… ****D. steposa**** (Ukraine)

## Discussion

Recently, Shvejonkova and Potapov (2011) , based mainly on published descriptions of species and their used names of genera, considered that “the independence of *Dimorphaphorura* calls for further ground.” For them, “several lines of Oligaphorurini independely undergo the reduction of furcal area, including furrow and number of manubrial seatae as well as the reduction of chaetotaxy of body and tibiotarsi, resulting in low value of these characters at level of generic taxonomy of Oligaphorurini.” They considered the independence of *Micraphorura* and *Oligaphorura* to be supported by the number and location of dental chaetae only, while shape of dental and manubrial area vary.

Shvejonkova and Potapov (2011) proposed a new tentative diagnosis for two of the main genera of the tribe Oligaphorurini, *Micraphorura* and *Oligaphorura* , both including species with or (more rarely) without anal spines as well as with different stages of furcal area development. For this reason, comparison of the furcal areas of *Oligaphorura* , *Micraphorura* , and *Dimorphaphorura* is presented in the *Remarks* to the genus *Dimorphaphorura* and in Figures 1–3 . For the moment it seems appropriate to preserve the genus *Dimorphaphorura* , whereas further investigations (including molecular sequencing) ould resolve problems related to the diagnosis of the three genera mentioned above.
